# Experimental Study on Reaction Characteristics of PTFE/Ti/W Energetic Materials under Explosive Loading

**DOI:** 10.3390/ma9110936

**Published:** 2016-11-18

**Authors:** Yan Li, Chunlan Jiang, Zaicheng Wang, Puguang Luo

**Affiliations:** State Key Laboratory of Explosion Science and Technology, Beijing Institute of Technology, Beijing 100081, China; 3120130104@bit.edu.cn (Y.L.); jiangchunwh@bit.edu.cn (C.J.); luopuguang1988@gmail.com (P.L.)

**Keywords:** reaction characteristics, energetic materials, explosive loading

## Abstract

Metal/fluoropolymer composites represent a new category of energetic structural materials that release energy through exothermic chemical reactions initiated under shock loading conditions. This paper describes an experiment designed to study the reaction characteristics of energetic materials with low porosity under explosive loading. Three PTFE (polytetrafluoroethylene)/Ti/W mixtures with different W contents are processed through pressing and sintering. An inert PTFE/W mixture without reactive Ti particles is also prepared to serve as a reference. Shock-induced chemical reactions are recorded by high-speed video through a narrow observation window. Related shock parameters are calculated based on experimental data, and differences in energy release are discussed. The results show that the reaction propagation of PTFE/Ti/W energetic materials with low porosity under explosive loading is not self-sustained. As propagation distance increases, the energy release gradually decreases. In addition, reaction failure distance in PTFE/Ti/W composites is inversely proportional to the W content. Porosity increased the failure distance due to higher shock temperature.

## 1. Introduction

Metal/fluoropolymer composites have been used in decoy flares for a long time. Now these composites are becoming a new category of energetic structural materials. Generally, they are formed by uniformly mixing active metal powders into a fluoropolymer matrix, followed by a pressing/sintering process. In contrast to traditional energetic materials, such as explosives and propellants, these materials are a class of solid energetic materials with higher mechanical strength and sufficient insensitivity [1,2]. Because they are inert under normal conditions, traditional initiation triggers such as flame or a detonator are not sufficient to initiate a reaction. However, under intense dynamic loading, a deflagration phenomenon can occur, in which the metal powder and fluoropolymer react together violently as a result of a shock-induced temperature rise. At the same time, a large amount of thermal energy and gases are released. Due to their mechanical strength and energetic characteristics, metal/fluoropolymer composites are widely used in both military and civil applications, including fragmentation warheads, shaped-charge warheads, penetrating warheads, and oil-well perforations [3,4]. During the process of penetration, the energetic components can not only penetrate the targets with their mechanical strength but also release chemical energy and expanding gases inside the targets. Compared with traditional inert materials, these energetic composites significantly enhance the structural damage inflicted on targets.

In recent years, researchers have made notable progress on shock-induced chemical reactions. Experimental approaches are commonly based on impact loading, including direct impact, indirect impact, and two-step impact [5]. McGregor [6] performed a plate impact experiment using a gas gun to determine the reaction onset time of highly porous PTFE (polytetrafluoroethylene)/Al mixtures. Ames [7], Mock [8,9], and Shen [10] studied the shock initiation of rods through Taylor impact tests, establishing the relationship between ignition delay time and impact energy. Wang [11] applied Hopkinson bar techniques to investigate the impact insensitivity of PTFE/Al/W composites with different W percentages, determining that the initial time, absorbed critical energy before the reaction, and incompleteness all exhibit clear increasing tendencies with increasing W content. Ames [12], Wang [13], Zhang [14], and Luo [15] used a vented chamber to measure the released energy of energetic fragments under various impact conditions, concluding that the extent of the reaction is influenced by impact velocity, strength properties, and target thickness.

Much previous work has documented ignition delay times and energy release efficiency. However, little attention has been paid to the reaction propagation in metal/fluoropolymer energetic materials under shock loading. Dolgoborodov [16] carried out experiments to explore combustion propagation in highly porous PTFE/Al mixtures initiated by low detonation velocity explosives, proving that it is possible to reach steady detonation. Nevertheless, the process by which metal/fluoropolymer composites with low porosity undergo reactions remains unknown.

The chemical reaction response of the shock wave in energetic materials is a complicated process involving shock-induced temperature rise and chemical reaction kinetics. This paper presents research on the reaction propagation of metal/fluoropolymer energetic materials with low porosity under explosive loading. PTFE/Ti/W composites with different W contents are investigated experimentally. Shock parameters are calculated based on experimental data, and differences in energy release are discussed.

## 2. Materials

### 2.1. Material Types

This study considers PTFE/Ti/W composites with mass ratios of 68/32/0, 34/16/50, 16/8/76, and 45/0/55. The relative mass ratio of PTFE to Ti for all composites is determined according to stoichiometry. Table 1 shows the mass ratios of the four kinds of PTFE/Ti/W composites, along with the corresponding theoretical maximum density (TMD), actual density, and relative density. It can be seen that the relative densities show obvious differences with changing mass ratios. Initially, the powders had the following average particle sizes: PTFE 34 μm, Ti 40 μm, and W 18 μm. The mixture of Ti and PTFE is a typical energetic structural material, in which Ti is oxidized by fluorine from PTFE. Ti reacts with PTFE promptly under impact conditions according to the following chemical equation:

Ti + (–C_2_F_4_–) → TiF_4_ (g) + 2C (s) + 892.5 kJ/mol (1)

Additional reactions are possible between the liberated carbon and ambient oxygen, which typically take place over much longer time scales [17]. Furthermore, additional energy is produced due to a phase change from gaseous TiF_4_ to solid TiF_4_. The additional reaction and phase change can be described as follows:

C (s) + O_2_ → CO_2_ + 393.5 kJ/mol (2)

TiF_4_ (g) → TiF_4_ (s) + 97.9 kJ/mol (3)

The addition of W powder increases the density of the energetic material without providing additional reactivity.

### 2.2. Preparation of PTFE (Polytetrafluoroethylene)/Ti/W Samples

**Figure 1:**
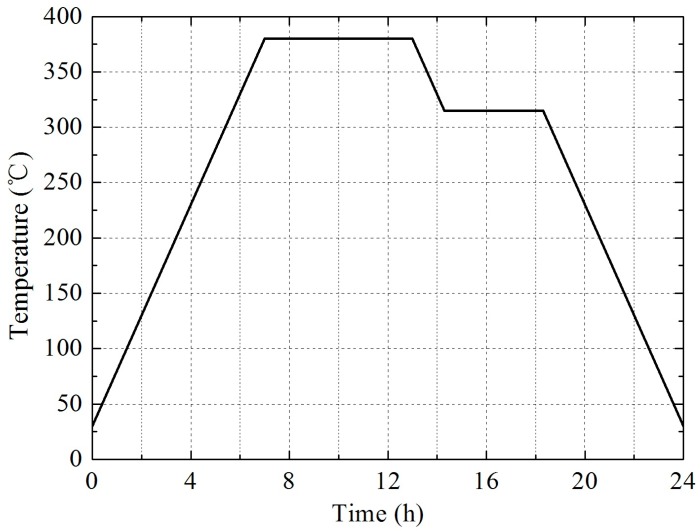
The temperature history of a sintering cycle.

**Figure 2:**
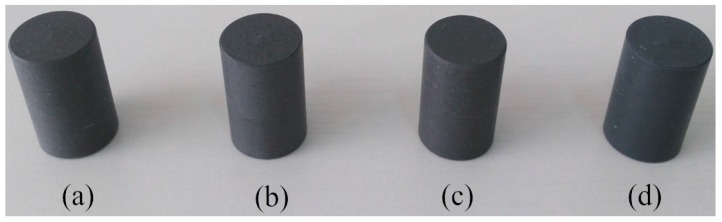
Sintered PTFE (polytetrafluoroethylene)/Ti/W samples: (**a**) 98.8% TMD 68PTFE/32Ti; (**b**) 90.3% TMD 34PTFE/16Ti/50W; (**c**) 70.7% TMD 16PTFE/8Ti/76W; and (**d**) 96.7% TMD 45PTFE/55W. TMD: theoretical maximum density.

The preparation process for the PTFE/Ti/W granular mixtures can be described as follows:

(1) First, the powders were mixed by a planetary mill machine (Chenli Powder Equipment Limited Company, Wuxi, China) for 24 h, with a small amount of absolute alcohol as a medium. Then, the powders were dried at 58 °C in a vacuum drying oven for approximately 24 h.
(2) The dried powder mixtures were pressed at 200 MPa for approximately 3 min through cold uniaxial pressing. Cylindrical samples with a size of φ20 × 30 mm were prepared.
(3) The samples were relaxed at ambient pressure and temperature for 24 h in order to remove trapped air and residual stress. The pressed samples were then sintered in an argon atmosphere with the temperature set at 380 °C. Figure 1 shows the temperature history of the sintering cycle, which can be described as follows: The oven temperature was raised up to 380 °C at a rate of about 50 °C/h. The samples were held at 380 °C for 6 h, after which the temperature was reduced at a rate of about 50 °C/h to 315 °C, where it was maintained for 4 h. The samples were then cooled to ambient temperature at an average cooling rate of 50 °C/h. The samples have little deformation during sintering due to the high melt viscosity of PTFE. The sintered PTFE/Ti/W samples are shown in Figure 2.

### 2.3. Microstructure of Composites

**Figure 3:**
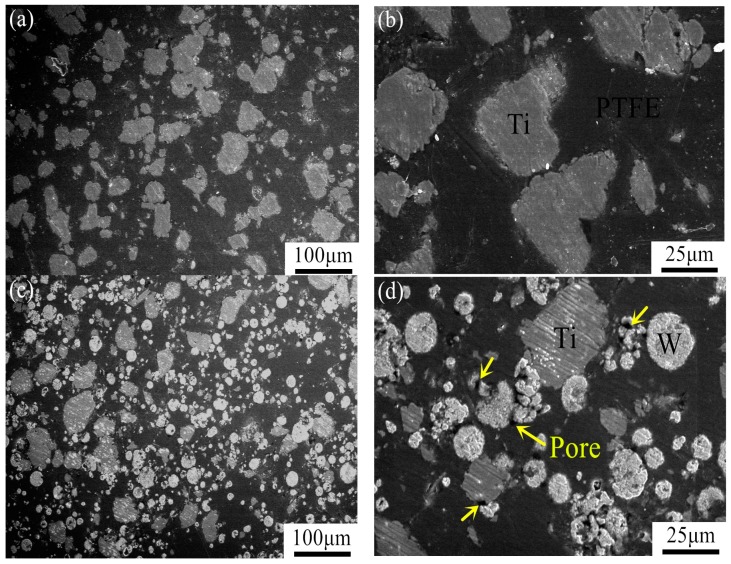

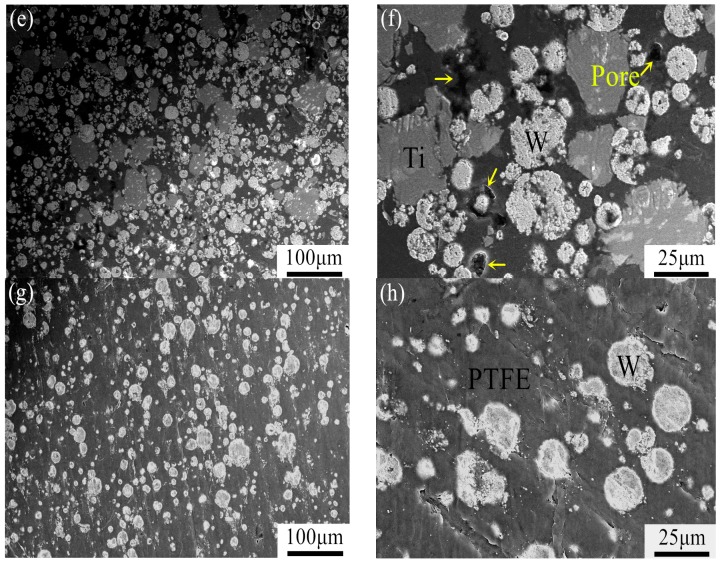
Backscattered scanning electron microscope (SEM) images of PTFE/Ti/W composites: (**a**,**b**) 98.8% TMD 68PTFE/32Ti; (**c**,**d**) 90.3% TMD 34PTFE/16Ti/50W; (**e**,**f**) 70.7% TMD 16PTFE/8Ti/76W; and (**g**,**h**) 96.7% TMD 45PTFE/55W.

Figure 3 shows the microstructures of the composites after sintering. These images demonstrate that the PTFE forms a continuous matrix in which W and Ti particles are discretely distributed. The components are labeled in the scanning electron microscope (SEM) images. As can be seen from the figure, the W particles are nearly spherical, but the shape of the Ti particles is irregular. The W particles are relatively bright because W has a high atomic number. The pores, which are noted by arrows, can be clearly seen in the 90.3% TMD 34PTFE/16Ti/50W and 70.7% TMD 16PTFE/8Ti/76Win high-magnification SEM images.

## 3. Experimental Methods

### 3.1. Experimental Technique

**Figure 4:**
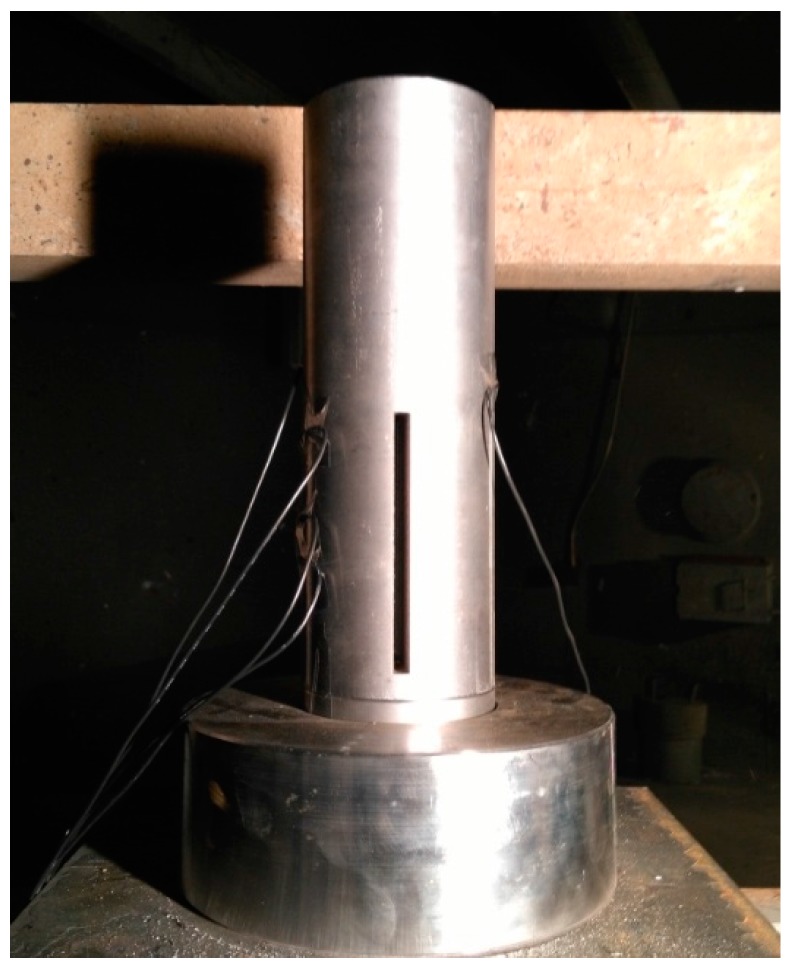
Photograph of experimental steel body.

**Figure 5:**
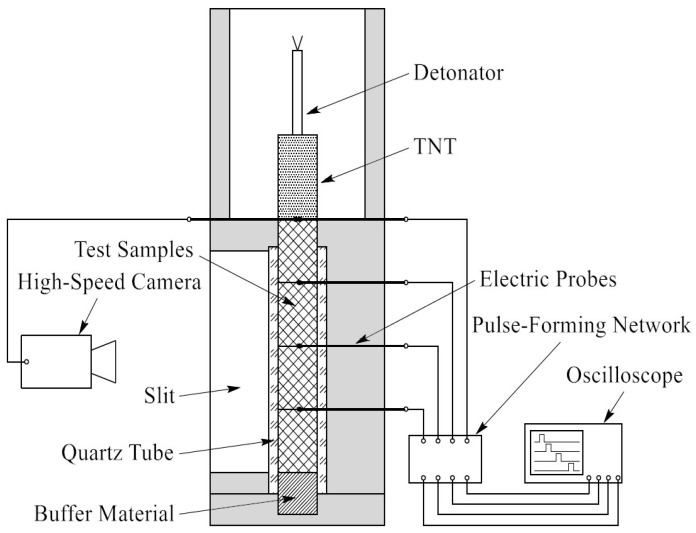
Schematic diagram of the experimental setup. TNT: trinitrotoluene.

The experiment was carried out in a cylindrical steel body containing a hollow structure, shown in Figure 4. Four samples were stacked vertically on the central axis, for a total height of approximately 120 mm. A transparent quartz tube with an inner diameter of 20 mm and thickness of 5 mm was installed between the samples and the steel body. On one side of the steel body, a slit was designed as an observation passageway through which the reaction process could be monitored. On the other side, four equally spaced holes were placed in the steel body and quartz tube, through which electric probes were inserted into the samples to half their diameters. On the top of the test samples, 20 g of pressed trinitrotoluene (TNT) was applied as an initiator to be triggered by an electric detonator. The sample of TNT with a size of φ20 × 40 mm is sufficient to make the incident shock front an approximately planar wave. The explosive was surrounded by high steel walls, so that the reaction light of the test samples would not be affected by the light produced by the TNT explosion. At the bottom of the samples, a buffer material made of 2024 aluminum alloy was installed to prevent intense impacts between the samples and the steel base. Figure 5 shows the schematic diagram of the experimental setup.

### 3.2. Test Methodology

When the shock wave propagates in the test samples, chemical reaction occurs after the shock front due to the shock-induced temperature rise. The shock front signal testing system is composed of electric probes, a resistance-inductance-capacitance (RLC) pulse-forming network, and an oscilloscope. Each probe is made by two fine enameled wires which have the diameter about 0.05 mm. These two wires are twined with each other and disconnected initially due to the insulation of enamel. When the shock wave propagates to the electric probe, the enamel will be damaged or melted instantaneously under the high pressure and temperature produced by the shock wave. The wires will be connected, and the RLC circuit will be conducted. An eight-volt electric pulse will be generated by capacitor discharge in RLC circuit, and the pulse signal will be displayed on the oscilloscope. Then the arrival time of shock front in the samples can be obtained according to the time of pulse’s rising edge. Simultaneously, when the shock front reaches the first electric probe, the high-speed camera is triggered, allowing the test samples’ reaction process under shock loading to be recorded through the observation window. This study set the image resolution to 384 × 80 pixels.

## 4. Results

**Figure 6:**
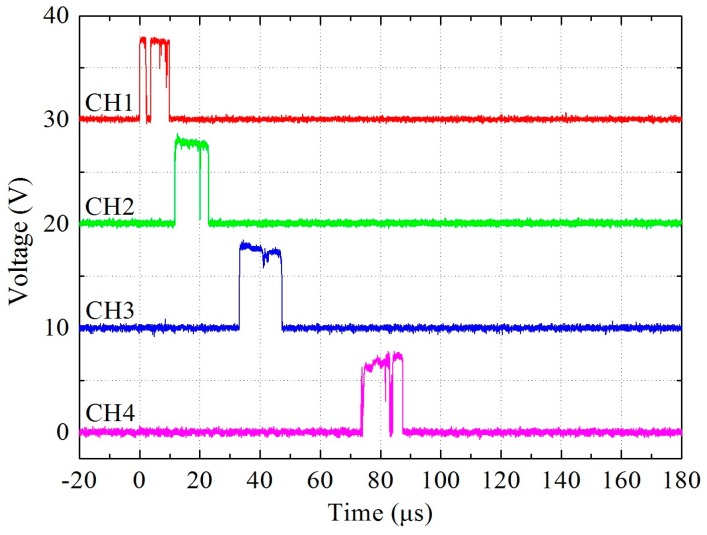
Typical shock front signals (Test No. 5).

**Figure 7:**
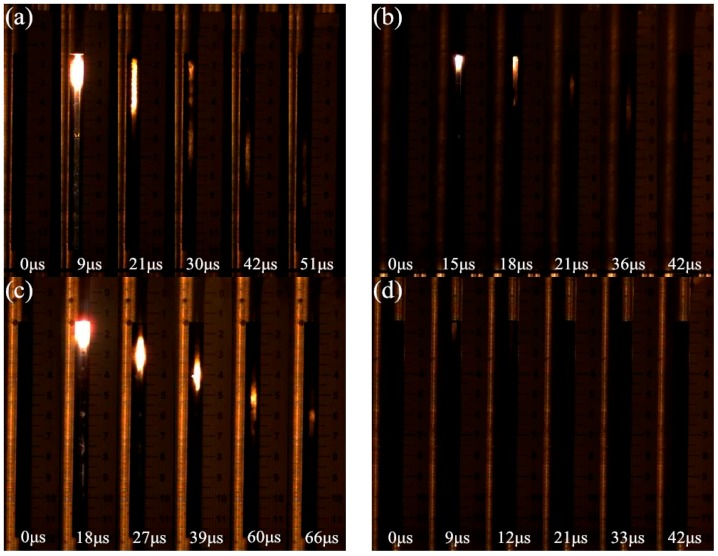
High-speed video sequences of partial tests. (**a**) Test No. 2; (**b**) Test No. 5; (**c**) Test No. 8; and (**d**) Test No. 10.

A series of explosive loading experiments were conducted. Table 2 summarizes data describing the time when the shock front reached the electric probes for each test. Figure 6 shows typical shock front signals as recorded by oscilloscope in test No. 5. The time intervals between neighboring signals increase progressively due to the attenuation of the shock wave. Figure 7 shows frames from high-speed video sequences of the tests. In the photographs of Ti-containing samples from tests No. 2, No. 5 and No. 8, a light propagates from top to bottom as a result of a shock-induced chemical reaction. In the beginning, the light is bright and the boundary is distinct; later, the light dims and the boundary blurs gradually. Finally, the light goes out. The sample that is 70.7% TMD 16PTFE/8Ti/76W produces a brighter initial light extending further down the sample, which shows a better reaction performance compared with others. A greater length of initial light is produced in sample 98.8% TMD 68PTFE/32Ti, but quickly dims. In contrast with the samples containing Ti, the PTFE/W sample (test No. 10) produces little light because of its inertness.

## 5. Discussion

### 5.1. Reaction Propagation under Explosive Loading

**Figure 8:**
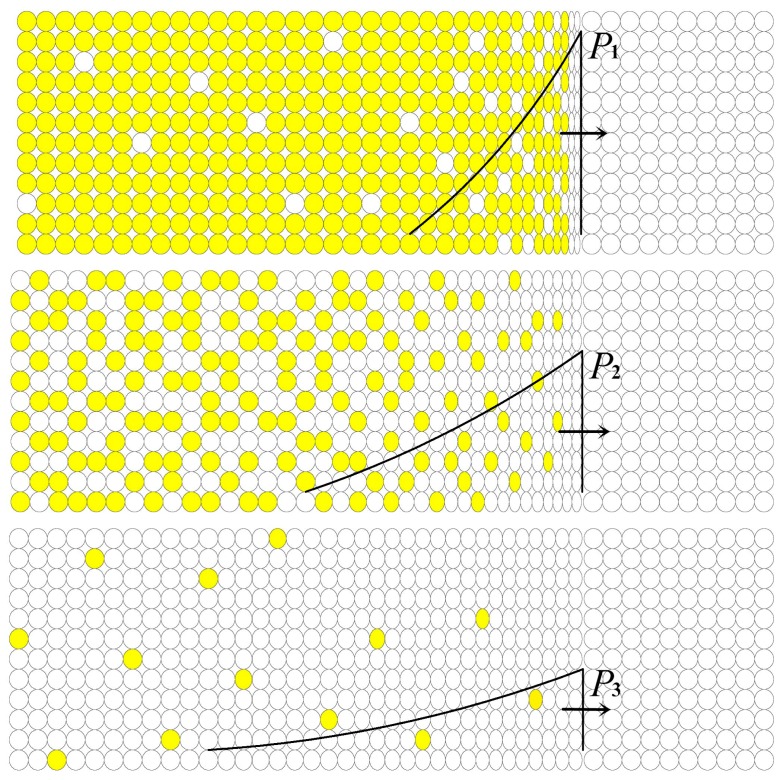
Schematic illustration of reaction propagation under explosive loading. The black profile represents the pulse shock wave propagating in the energetic materials. The arrow shows the propagation direction. The use of color within the idealized microstructure symbolizes the chemical reaction.

This section describes the reaction propagation of PTFE/Ti/W energetic materials with low porosity under explosive loading, with a corresponding schematic illustration shown in Figure 8.

When detonation wave of TNT reaches the interface between the explosive and the energetic test samples, a triangular shock pulse with initial peak pressure *P*_1_ generates and propagates into the test samples. As the shock-induced instantaneous temperature rises, a chemical reaction occurs shortly behind the shock front [18]. Initially, a high temperature rise is expected due to the intense shock compression, which induces a prompt and powerful chemical reaction between Ti and PTFE. As a result, a bright light with a distinct boundary can be observed.

**Figure 9:**
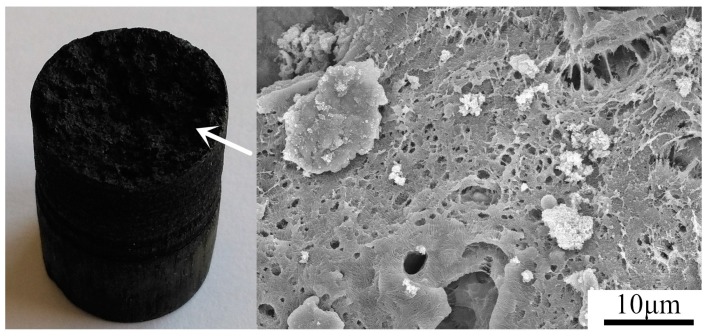
Residual sample and SEM micrographs of reaction surface after test No. 2. The arrow indicates the reaction surface.

As the shock wave propagates, the waveform changes gradually. The peak pressure decreases to *P*_2_, and the pulse duration increases due to the rarefaction wave following the shock front. The rarefaction wave takes energy away from the shock pulse. The chemical reaction energy has no sufficient rapidity or quantity to balance the energy losses of rarefaction wave, resulting in a reduction of the shock-induced temperature rise and therefore decreasing the extent of reaction between Ti and PTFE. Less reactant is converted into product, and the reaction is not as prompt as in the initial state. Consequently, the light dims and the boundary becomes blurred. Figure 9 depicts a residual sample after test No. 2. Microscopically, the reaction surface is rough and porous, providing direct evidence of the incomplete reaction.

As the peak pressure of the shock pulse attenuates to *P*_3_, the pulse duration further increases and shock temperature further decreases. At this time, the reaction occurs only at a few points, and the reaction is so weak that little light can be observed. Finally, the light goes out because of the further attenuation of the shock wave.

These results demonstrate that the reaction propagation of PTFE/Ti/W energetic materials with low porosity is not self-sustained. The energy released by the chemical reaction is insufficient to compensate for energy loss caused by the shock attenuation. The shock-induced chemical reaction is very sensitive to the shock pressure, and the energy release is directly related to shock temperature. Accordingly, it is necessary to calculate the shock parameters under explosive loading for different energetic samples.

### 5.2. Theoretical Calculation of Shock Parameters

This section presents a theoretical calculation of shock parameters based on experimental data, from which the shock pressure and shock temperature are obtained for different samples.

**Figure 10:**
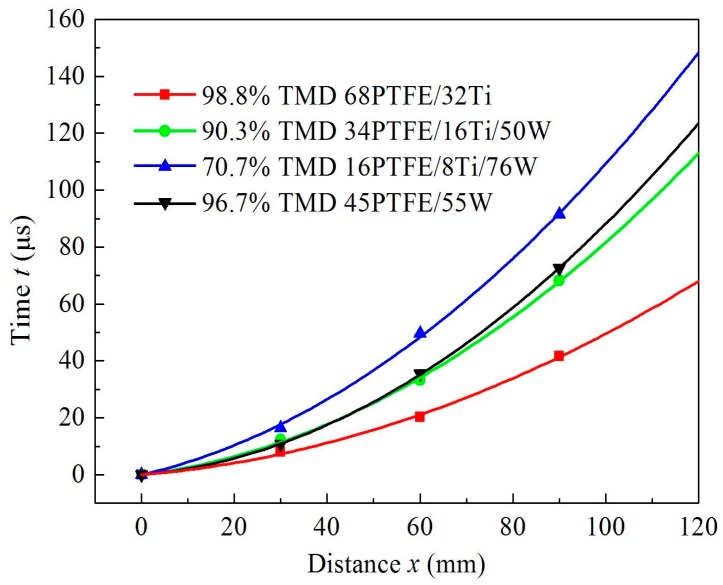
Time–distance curves for shock wave propagation.

The time when shock front reaches each electric probe can be averaged for samples with the same material proportions, and a second-order polynomial can be used to fit a time–distance relationship based on the least squares theory. The fitting curve can be expressed as follows:

(4) $$t=ax^{2}+bx$$

where *t* and *x* are the time and distance, respectively, at which the shock front reaches the electric probes; and *a* and *b* are fitting coefficients, which are listed in Table 3. The fitting curves for different samples are shown in Figure 10. Then the shock wave velocity can be expressed as:

(5) $$U_{S}=\frac{dx}{dt}=\frac{1}{\sqrt{b^{2}+4at}}=\frac{1}{2ax+b}$$

**Figure 11:**
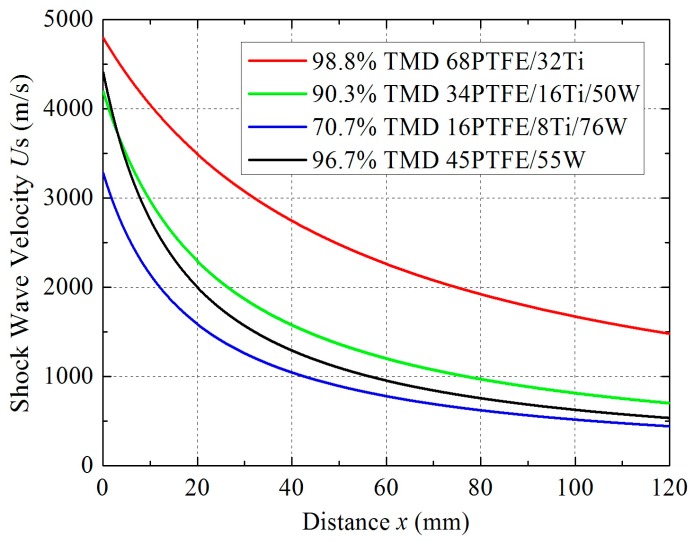
Velocity-distance curves for shock wave propagation.

The *U*_S_*–x* curves for different samples are presented in Figure 11.

McQueen [19] derived a popular equation of state (EOS) for porous materials:

(6) $$P=\frac{\left[2V-\gamma \left(V_{0}-V\right)\right]C^{2}\left(V_{0}-V\right)}{\left[2V-\gamma \left(V_{00}-V\right)\right]{\left[V_{0}-S\left(V_{0}-V\right)\right]}^{2}}$$

where *P* is the shock wave pressure; *C*, *S*, and $V_{0}=1/\rho_{0}$ are the longitudinal elastic wave velocity at zero pressure, empirical Hugoniot coefficient, and specific volume of the fully dense solid with TMD, respectively; and γ, *V*_00_, and *V* are the Grüneisen coefficient, initial specific volume, and post-shock specific volume of the porous material, respectively.

The following expression is an approximate equation that can be used based on the assumption that γ/V is a constant [20]:

(7) $$\frac{\gamma }{V}=\frac{\gamma_{0}}{V_{0}}=\mathrm{const}$$

The parameters *C*, *S*, and $\gamma_{0}$ can be estimated from the experimental data of the constituent materials by mass-weighted average method, as follows:

$$C=\sum m_{i}C_{i},\;S=\sum m_{i}S_{i},\;\gamma_{0}=\sum m_{i}\gamma_{0i}$$

where *m_i_* is the mass fraction of constituent materials, the parameters of which are listed in Table 4.

According to the one-dimensional shock wave theory, the mass conservation equation and momentum conservation equation for porous materials under shock loading can be written as follows:

(8) $$V_{00}\left(U_{S}-U_{P}\right)=VU_{S}$$

(9) $$V_{00}P=U_{S}U_{P}$$

where $U_{P}$ is the post-shock particle velocity. Substituting Equation (9) into Equation (8), pressure can be derived as:

(10) $$P=\frac{U_{S}{}^{2}\left(V_{00}-V\right)}{V_{00}{}^{2}}$$

**Figure 12:**
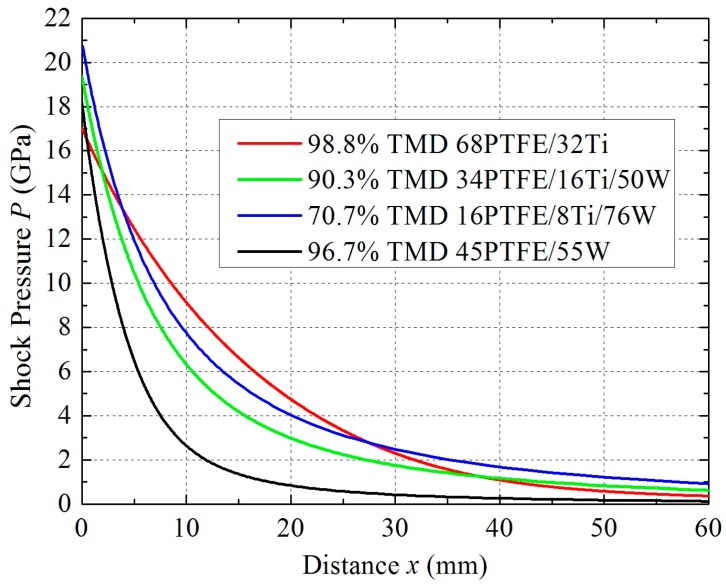
Pressure–distance curves for shock wave propagation.

Combining Equations (5), (6), and (10) can establish the relationship between pressure *P* and distance *x* of shock wave propagation. Figure 12 presents the *P–x* curves for different samples. As Figure 12 shows, the initial pressure varies from 17 to 20.7 GPa due to differences in wave impedance. The 70.7% TMD 16PTFE/8Ti/76W has the highest initial pressure, since it has the highest actual density. On the contrary, the initial pressure is the lowest in 98.8% TMD 68PTFE/32Ti because of its lowest actual density. The 70.7% TMD 16PTFE/8Ti/76W does not show a great reduction in pressure because its greater reactivity compensates for the effect of higher actual density and porosity. In contrast, the shock pressure in sample 96.7% TMD 45PTFE/55W attenuates the most rapidly as a result of its inertness.

When the shock wave propagates in the test samples, the material is compressed and experiences an instantaneous temperature rise. The thermodynamic process at the shock front is assumed to be adiabatic. The energy conservation equation for the Hugoniot shock process is written as follows:

(11) $$E-E_{0}=\frac{1}{2}\left(P+P_{0}\right)\left(V_{00}-V\right)$$

where *E* is specific internal energy along the Hugoniot curve; and *E*_0_ and *P*_0_ refer to initial internal energy and pressure, respectively. When *P*_0_ equals zero, the shock temperature *T* can be expressed by the following equation based on the assumption that the change of internal energy is completely converted into shock temperature,

(12) $$T=T_{0}+\frac{P\left(V_{00}-V\right)}{2C_{\text{v}}}$$

where *T*_0_ is ambient initial temperature, and *C*_v_ is the heat capacity at constant volume, which can be estimated by mass-weighted average method,

$$C_{v}=\sum m_{i}C_{vi}$$

**Figure 13:**
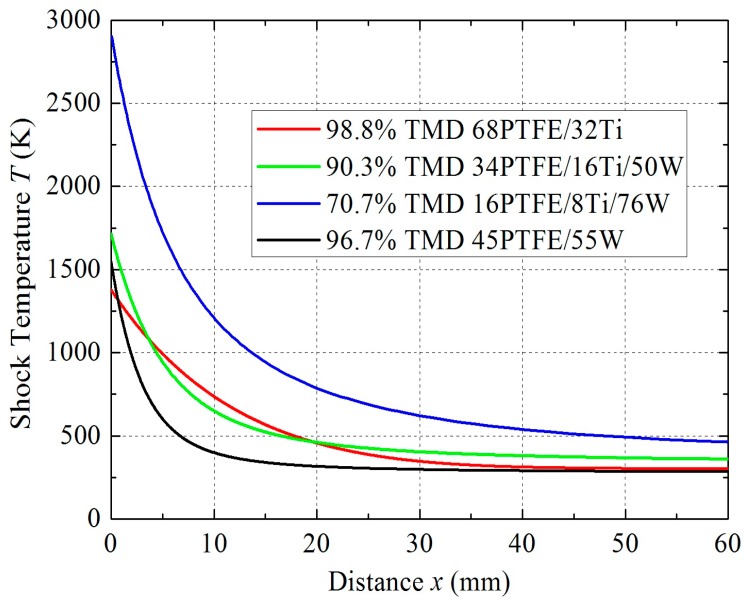
Temperature–distance curves for shock wave propagation.

Figure 13 presents the relationship between shock temperature *T* and distance *x* for different samples. As illustrated in the *T–x* space, the temperature decreases rapidly as a result of shock pressure reduction. The temperature rise is more obvious in 70.7% TMD 16PTFE/8Ti/76W than in other materials because of its relatively high porosity. The temperature decreases the most rapidly in 96.7% TMD 45PTFE/55W due to its inertness.

### 5.3. Analysis of Differences in Energy Release

The shock-induced chemical reaction process is assumed to be temperature-controlled. The energy release *q* can be characterized by the following equation:

(13) q=N⋅y⋅ΔH

where *N* is the molar number of the reactant per unit volume, *y* is the reaction extent, which is related to the shock temperature, and ΔH is the heat release per mole of reactant. Table 5 lists the molar numbers of reactants per unit volume for different samples.

**Figure 14:**
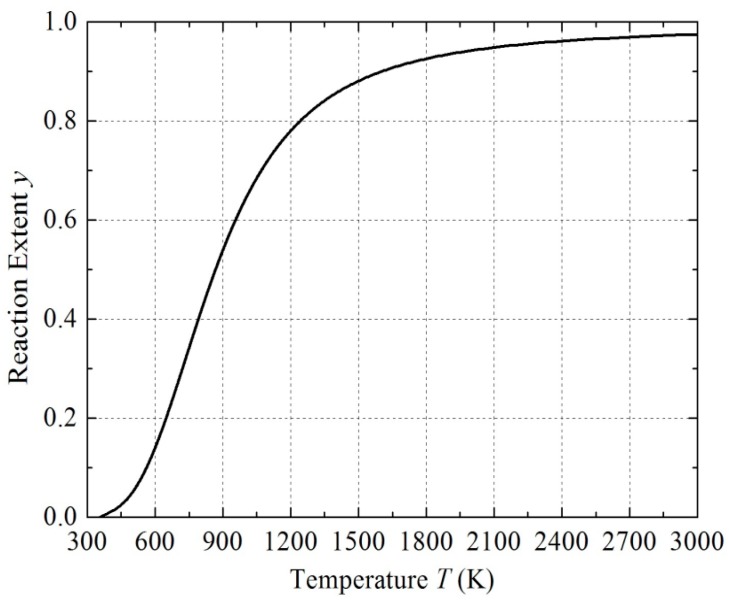
Curve of reaction extent changing with temperature for PTFE/Ti.

The reaction extent can be calculated through chemical reaction kinetics. The Avrami–Erofeev equation is popularly used to describe solid state reactions under a high rate of temperature rising, the equation is expressed as follows [21]:

(14) $$\frac{dT}{dy}=\frac{RT^{2}}{E_{a}}\left[\frac{1}{2y}-\frac{n\ln\left(1-y\right)+n-1}{n(1-y)[-\ln(1-y)]}\right]$$

where *T* is absolute temperature, *E_a_* is apparent activation energy, *R* is molar gas constant, and *n* is a coefficient related to boundary conditions and reaction mechanisms. The reaction parameters of PTFE/Ti are tabulated in Table 6 and the *y–T* curve of PTFE/Ti is shown in Figure 14.

**Figure 15:**
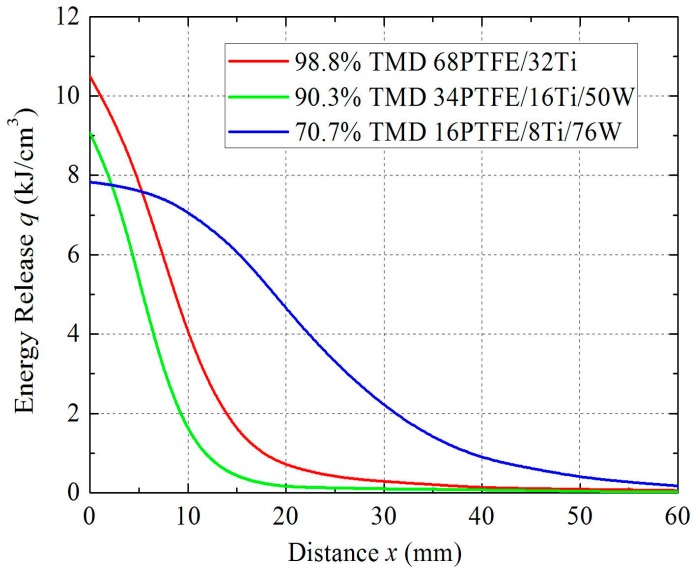
Curves of energy release changing with distance.

Then, the relationship between energy release *q* and distance *x* can be established for different samples, as shown in Figure 15.

As Figure 15 shows, all samples release a relatively large amount of energy at the beginning because of the high temperature rise. As the propagation distance increases, the temperature gradually decreases. As a result, the reaction extent decreases, which is reflected by reductions in brightness. Finally, the light goes out, indicating that the energy release has decreased almost to zero.

Compared with 98.8% TMD 68PTFE/32Ti, 90.3% TMD 34PTFE/16Ti/50W experiences a similar shock temperature. However, the quantity of reactant decreases when the inert tungsten powder is added, leading to a decrease in energy release per unit volume. In addition, the W absorbs some of the energy released by the reaction in heating up the inert W particles, which will further reduce the energy. Consequently, the reaction produces a relatively dim light.

Compared with 90.3% TMD 34PTFE/16Ti/50W, the quantity of reactant for 70.7% TMD 16PTFE/8Ti/76W is even further decreased, and the W further absorbs the energy released by the reaction. However, the shock temperature is more dramatic due to the significant increase in porosity, leading to a higher energy release. Therefore, the reaction produces a brighter light with a longer continuous distance.

In summary, the results of these experiments demonstrate that the energy release of PTFE/Ti/W composites under explosive loading is influenced by propagation distance, W content, and porosity.

## 6. Conclusions

This paper described experimental research and theoretical analysis of the reaction characteristics of PTFE/Ti/W energetic materials under explosive loading. The process of shock-induced chemical reaction was described, and differences in energy release were discussed based on shock parameters calculation. The following specific conclusions can be drawn:

(1) The reaction propagation of PTFE/Ti/W energetic materials with low porosity under explosive loading is not self-sustained. Chemical reaction occurs shortly behind the shock front. The energy released during reaction is not sufficient to overcome the attenuation of the shock. The extent of the reaction depends on the shock energy, which is reflected by shock pressure and shock temperature. Owing to the attenuation of shock energy during propagation, the reaction extent decreases rapidly.
(2) Propagation distance, W content, and porosity all influence the energy release of PTFE/Ti/W composites under explosive loading. As the propagation distance increases, the energy release shows a decreasing tendency due to temperature reductions. Moreover, the addition of inert W powder reduces the quantity of reactant as well as provides a heat sink, causing a decrease in overall energy release. Porosity plays a significant role in energy release: shock temperature increases as porosity improves, leading to remarkable increases in energy release.
(3) The 70.7% TMD 16PTFE/8Ti/76W is more reactive under explosive loading, followed by 98.8% TMD 68PTFE/32Ti and 90.3% TMD 34PTFE/16Ti/50W.

## Figures and Tables

**Table 1:** Material properties of experimental samples. PTFE: polytetrafluoroethylene; TMD: theoretical maximum density.

Materials	TMD (g/cm^3^)	Density (g/cm^3^)	Relative Density
PTFE/Ti/W (68/32/0)	2.59	2.56	98.8%
PTFE/Ti/W (34/16/50)	4.57	4.13	90.3%
PTFE/Ti/W (16/8/76)	7.57	5.35	70.7%
PTFE/Ti/W (45/0/55)	4.20	4.06	96.7%

**Table 2:** Test results.

Number	Materials	*t*_1_ (μs) ^a^	*t*_2_ (μs) ^a^	*t*_3_ (μs) ^a^
1	PTFE/Ti/W (68/32/0)	8.1	20.3	40.5
2	PTFE/Ti/W (68/32/0)	8.7	19.8	38.8
3	PTFE/Ti/W (68/32/0)	8.3	21.4	41.6
4	PTFE/Ti/W (34/16/50)	12.3	31.7	69.8
5	PTFE/Ti/W (34/16/50)	11.8	33.2	73.6
6	PTFE/Ti/W (34/16/50)	13.0	34.3	70.2
7	PTFE/Ti/W (16/8/76)	16.5	49.3	88.2
8	PTFE/Ti/W (16/8/76)	17.2	52.0	93.1
9	PTFE/Ti/W (16/8/76)	15.4	50.8	91.5
10	PTFE/Ti/W (45/0/55)	11.6	31.5	68.6

^a^ *t*_1_, *t*_2_, and *t*_3_ are the time of the shock wave’s propagation from the first electric probe to the second, the third, and the fourth probe, respectively.

**Table 3:** Fitting coefficients of different experimental samples.

Materials	*a*	*b*
PTFE/Ti/W (68/32/0)	1.95 × 10^−3^	2.08421 × 10^−4^
PTFE/Ti/W (34/16/50)	4.96 × 10^−3^	2.37858 × 10^−4^
PTFE/Ti/W (16/8/76)	8.17 × 10^−3^	3.03756 × 10^−4^
PTFE/Ti/W (45/0/55)	6.86 × 10^−3^	2.25798 × 10^−4^

**Table 4:** Parameters of constituent materials.

Material	$\rho_{0}$ (g/cm^3^)	*C* (m/s)	*S*	$\gamma_{0}$	*C*_v_ (J/g·K)
PTFE	2.150	1682	1.819	0.59	1.048
Ti	4.528	5220	0.767	1.09	0.519
W	19.224	4029	1.237	1.54	0.131

**Table 5:** Molar numbers of reactants per unit volume. *N*: molar number of the reactant per unit volume.

Materials	*N* (mol/L)
98.8% TMD 68PTFE/32Ti	17.31
90.3% TMD 34PTFE/16Ti/50W	13.96
70.7% TMD 16PTFE/8Ti/76W	8.68

**Table 6:** Reaction parameters.

Material	Δ*H* (kJ/mol)	*E*_a_ (kJ/mol)	*n*
PTFE/Ti (67.6/32.4)	893	64.8 ^a^	0.25 ^b^

^a^ *E*_a_ is determined through differential scanning calorimetry (DSC) experiments; ^b^ Qiao, L. [22].
